# Sleep and circadian parameters in Behçet’s syndrome: a comparative analysis using actigraphy and questionnaires

**DOI:** 10.1093/rheumatology/keaf326

**Published:** 2025-06-09

**Authors:** Alessandro Colitta, Simone Bruno, Francy Cruz-Sanabria, Federico Starace, Andrea Bazzani, Federica Di Cianni, Paolo Frumento, Michelangelo Maestri Tassoni, Enrica Bonanni, Marta Mosca, Rosaria Talarico, Ugo Faraguna

**Affiliations:** Department of Clinical and Experimental Medicine, Neurology Unit, University of Pisa, Pisa, Italy; Department of Psychiatry, University of Wisconsin-Madison, Madison, WI, USA; Department of Translational Research and of New Surgical and Medical Technologies, University of Pisa, Pisa, Italy; Department of Developmental Neuroscience, Istituto di Ricovero e Cura a Carattere Scientifico (IRCCS) Foundation Stella Maris, Pisa, Italy; Department of Translational Research and of New Surgical and Medical Technologies, University of Pisa, Pisa, Italy; Institute of Management, Sant’Anna School of Advanced Studies, Pisa, Italy; Department of Clinical and Experimental Medicine, Rheumatology Unit, University of Pisa, Pisa, Italy; Department of Political Sciences, University of Pisa, Pisa, Italy; Department of Clinical and Experimental Medicine, Neurology Unit, University of Pisa, Pisa, Italy; Department of Clinical and Experimental Medicine, Neurology Unit, University of Pisa, Pisa, Italy; Department of Clinical and Experimental Medicine, Rheumatology Unit, University of Pisa, Pisa, Italy; Department of Clinical and Experimental Medicine, Rheumatology Unit, University of Pisa, Pisa, Italy; Department of Translational Research and of New Surgical and Medical Technologies, University of Pisa, Pisa, Italy; Department of Developmental Neuroscience, Istituto di Ricovero e Cura a Carattere Scientifico (IRCCS) Foundation Stella Maris, Pisa, Italy

**Keywords:** Behçet disease, fibromyalgia, quality of life, nervous, rare diseases, vasculitis, chronic pain syndromes

## Abstract

**Objective:**

Sleep disturbances are highly prevalent in Behçet syndrome (BS) patients. Within this population, sleep disturbances are frequently associated with active disease and comorbid fibromyalgia. However, possible sleep impairments in BS patients without these conditions remain poorly explored, along with BS patients’ obstructive sleep apnoea syndrome (OSAS) risk and circadian rhythm preferences. We aimed to address these research gaps through a cross-sectional study comparing sleep and circadian parameters between BS patients, with or without active disease and comorbid fibromyalgia, and healthy controls (HCs).

**Methods:**

Participants’ sleep and circadian parameters were evaluated objectively via actigraphy and subjectively through the Pittsburgh Sleep Quality Index and the reduced Morningness–Eveningness Questionnaire. A comprehensive clinical evaluation investigated sociodemographic data, disease activity and comorbid fibromyalgia. Possible predictors of sleep and circadian parameters were tested estimating linear regression models.

**Results:**

Forty-five BS patients and 61 age-, BMI-, sex- and smoking habits-matched HCs were enrolled. Only BS patients with active disease and/or fibromyalgia showed significantly lower sleep quality, significantly higher sleep fragmentation and a tendence towards less robust circadian rhythms compared to other participants. Instead, BS patients without those conditions did not significantly differ from HCs in sleep and circadian parameters. Furthermore, a higher actigraphically-determined OSAS risk was found in all BS patients compared to HCs.

**Conclusions:**

Active disease and fibromyalgia are associated with sleep and circadian rhythm disturbances in BS patients. Screening for sleep and circadian rhythm disturbances may be advised in BS patients with these conditions, while OSAS screening may be recommended in all BS patients with sleep disturbances.

Rheumatology key messages

- Fibromyalgia and active disease are key correlates of BS patients’ sleep disturbances and circadian typology.
- BS patients without active disease or fibromyalgia and healthy controls show comparable rest–activity rhythms.
- All BS patients show higher obstructive sleep apnoea syndrome risk compared to HCs.

## Introduction

Behçet syndrome (BS) is a rare, chronic, systemic vasculitis that severely affects patients’ quality of life (QoL) [1]. Impaired sleep is one of the primary factors impacting QoL [1, 2], affecting up to 73.6% of BS patients [1]. Within this population, moreover, active disease and fibromyalgia commonly coexist with sleep disturbances [1, 3, 4]. In a previous study investigating clinical correlates of BS patients’ sleep disturbances, we found a 43% prevalence of poor sleep quality among non-active, non-fibromyalgic BS patients (median age = 50; IQR = 43.75–52.25), as measured by the Pittsburgh Sleep Quality Index (PSQI) [5]. This finding aligns with the previously characterized prevalence of sleep disturbances in age-matched individuals from the general population [6, 7], suggesting that non-active, non-fibromyalgic BS patients and the general population may report similar sleep quality. Notably, no study compared sleep and circadian parameters between non-active, non-fibromyalgic BS patients and individuals without BS [1]. Confirming this hypothesis would corroborate the previously suggested role of disease activity and fibromyalgia in BS patients’ sleep impairment, potentially supporting the integration of sleep disturbances screening in BS populations at higher risk of developing sleep impairments.

In BS patients, sleep has been mainly investigated through self-report scales [1], with the PSQI being the most widely used [1]. Furthermore, objective assessment of BS patients’ sleep has been employed only in three studies [5, 8, 9], with only one study characterizing sleep and circadian rhythms through actigraphy [5], a non-invasive sleep–wake assessment method which can provide quantitative sleep and circadian parameters in ecological settings based on relatively long-term activity monitoring [10–12]. Nonetheless, no study has yet compared actigraphic parameters between BS patients and healthy individuals [1, 5, 13], leaving possible differences in objective sleep and circadian parameters unexplored in ecological settings, where sleep parameters are not influenced by changes in the sleep environment [14].

Recent literature focused on circadian rhythm characterization in immune-mediated diseases, with focus on self-reported circadian typology (i.e. chronotype) [13, 15]. A recent systematic review showed that eveningness, which refers to the preference for later sleep–wake schedule timings, is associated with higher patient-reported symptoms burden within this population, despite no significant associations with disease activity [13]. Nevertheless, to the best of our knowledge, no study compared self-report and objective circadian parameters between BS patients and healthy individuals [1, 13, 16].

Limited research explored obstructive sleep apnoea syndrome (OSAS) risk in BS with partially inconsistent results [1, 8, 9, 17, 18]. A large population study suggested that BS patients longitudinally show a higher risk of developing OSAS compared to individuals without autoimmune disorders [18]. However, the two groups were not matched for BMI [19], a key risk factor for OSAS development [20]. In parallel, two cross-sectional sleep laboratory studies employing polysomnography showed a higher respiratory disturbance index in BS patients compared to the controls [8, 9]. In partial contrast, a cross-sectional study by Gokturk *et al.* demonstrated that only BS patients suffering from superior vena cava syndrome (SCVS) show significantly higher OSAS risk compared to both BS patients without SCVS and healthy controls (HCs), as measured by the Berlin questionnaire [17]. Given these partially inconsistent findings [8, 9, 17, 18], further studies should confirm possible differences in OSAS risk between BS patients and healthy individuals, accounting for possible confounding factors such as age, sex, BMI and smoking habits [19]. Of note, a novel machine-learning algorithm estimating OSAS risk through actigraphic data has been recently validated [12]; however, this method remains to be applied in BS patients [1, 8, 9, 17, 18].

Within this framework, we aimed at: (i) comparing actigraphic and self-reported sleep and circadian parameters between BS patients and HCs; (ii) exploring possible differences in sleep and circadian parameters between HCs and two cohorts of BS patients (i.e. BS patients with active disease and/or fibromyalgia, and BS patients without those conditions); (iii) comparing OSAS risk between the BS patients and the HCs through a novel machine-learning algorithm using actigraphic data [12].

## Materials and methods

### Eligibility criteria and ethics statement

Adult participants fulfilling the following inclusion criteria were recruited: provided the signed informed consent; being fluent in Italian; not being a shift worker. BS patients additionally met the International Study Group for Behçet Disease Criteria [21]. Individuals diagnosed with sleep disorders or rheumatologic, neurologic and psychiatric diseases were not included among HCs. The study was approved by the Bioethical Committee of the University of Pisa (19233_MOSCA) and was conducted in compliance with the principles of the Declaration of Helsinki.

### Study design and procedure

A cross-sectional study was conducted. BS patients were consecutively recruited during their routine follow-up visits at the Behçet Clinic of the University Hospital of Pisa, when they underwent a comprehensive clinical evaluation investigating eligibility criteria (2025) [5], disease activity [22], possible presence of fibromyalgia [23], pharmacological history. Age-, BMI-, sex- and smoking habits-matched HCs were consecutively recruited after a clinical interview investigating their eligibility criteria.

At the end of the eligibility criteria screening, participants were given both an actigraph, to be continuously worn for a week, and a digital survey combining self-report questionnaires and basic questions on sociodemographic data. Self-report questionnaires explored participants’ sleep quality and chronotype. After the visits, medical records were systematically examined to gather information about BS patients’ exposure to glucocorticoids (GC) and history of organ involvement.

### Measures

#### Sociodemographic and clinical data

The following sociodemographic data were collected: age, BMI, sex, smoking habits (smoker yes/no, number of cigarettes per week) and shift working (yes/no). BS disease activity was evaluated using the Behçet Disease Clinical Activity Form (BDCAF). The BDCAF evaluates BS disease activity over the previous 30 days through 12 weighted elements [22]. The cut-off score for active disease was BDCAF ≥ 2 based on previous literature [5, 24]. Fibromyalgia was diagnosed based on the Analgesic, Anesthetic and Addiction Clinical Trial Translations Innovations Opportunities and Networks–American Pain Taxonomy Diagnostic Criteria [23]. Furthermore, the following clinical information was gathered: history of organ involvement, immunosuppressive and immunomodulatory treatment (yes/no; drug name), current treatment for mood disorders and/or insomnia (yes/no; drug type, included over the counter medications) and GC exposure variables, that is current GC use (yes/no), daily oral GC dose (mg/day, prednisone equivalents) [5, 25] and one-year cumulative GC dose, defined as the total amount of GC administered to a patient over the previous year disease duration (mg, prednisone equivalents) [5].

#### Self-report questionnaires

The PSQI is a self-administered 19-item questionnaire assessing sleep quality over a one-month time interval [26]. The sum of the score ranges from 0 to 21; a global score >5 is an index of poor sleep quality, defining an individual as a poor sleeper [26]. The reduced Morningness–Eveningness Questionnaire (rMEQ) is the reduced version of the MEQ, a self-administered, validated questionnaire to estimate the chronotype of an individual, validated in Italian language [27]. The total score ranges from 4 to 26: according to the scale, a score <11 indicates an evening type; 11–18 a neither type; >18 a morning type.

#### Actigraphy

All subjects continuously wore a Fitbit Inspire 2^®^ (FI2) for seven days on their non-dominant wrist. The FI2 smartband is a commercial device capable of tracking wrist activity with a 1-min resolution through a micro-electro-mechanical systems triaxial motion sensor and heart rate data through a photoplethysmographic sensor (PurePulse^®^ light-emitting diode). Rest–activity data were digitally stored for subsequent analysis through the Dormi algorithm by Sleepacta s.r.l., a validated artificial neural network-based script [28] registered as a medical, risk class I device within the European Database on Medical Devices (EUDAMED) (UDI-DI: PP13374SLEEP78/IFA). The following quantitative actigraphic sleep parameters were estimated through the Dormi algorithm [5, 10, 11, 25]: total sleep time (TST), waking after sleep onset (WASO), sleep efficiency (SE), sleep regularity index (SRI), mid-sleep point.

To estimate parametric rest–activity circadian rhythm parameters, that is Midline-Estimating Statistic of Rhythm (MESOR), amplitude and acrophase [29], we applied the cosinor method by fitting a sine wave to the acquired actigraphic data through the R package ‘Cosinor’ [30]. Non-parametric measurements of rest–activity rhythms, that is interdaily stability (IS), intradaily variability (IV) and relative amplitude (RA) [31] were computed using the R package Non-Parametric Measures of Actigraphy Data ‘nparACT’ [32]. A detailed description of estimated accelerometric sleep and circadian parameters is reported in Supplementary Data S1, available at *Rheumatology* online.

Pre-test actigraphic risk of OSAS was estimated through DormiApnea, a validated machine-learning algorithm registered as a medical, risk class I device within the EUDAMED (UDI-DI: PP13374SLEEP78/IFA). DormiApnea employs accelerometric activity, sleep parameters, heart rate data, age, sex and BMI [12] to categorize participants into four pre-test OSAS risk groups, that is ‘Low’, ‘Mild’, ‘Moderate’, ‘High’, which correspond to different levels of estimated OSAS severity, in line with standard Apnoea-Hypopnoea Index (AHI) classifications [12]. In detail, ‘Low’ risk suggests the absence of OSAS (AHI < 5), ‘Mild’ risk indicates possible presence of mild OSAS (5 ≤ AHI < 15), ‘Moderate’ risk corresponds to moderate OSAS (15 ≤ AHI < 30) and ‘High’ risk to severe OSAS (AHI ≥ 30) [12].

### Data analysis

Data were analysed in RStudio 4.1.1. Median and interquartile range (IQR) described quantitative variables, a table of frequencies and percentages presented categorical variables.

First, non-parametric tests were used to test possible differences in sleep and circadian parameters between BS patients and HCs. Secondly, participants were divided into three sub-cohorts (i.e. HCs, BS patients with active disease and/or fibromyalgia, and BS patients without those conditions), and possible differences in sleep and circadian parameters between the three sub-cohorts were subsequently tested. Furthermore, *post hoc* tests were performed to explore possible differences in sleep and circadian parameters between couples of sub-cohorts (e.g. HCs and active and/or fibromyalgic BS patients).

Multiple linear regression models were computed to confirm the results from non-parametric tests, including age, BMI, sex, smoking habit (yes/no) and treatment for mood disorders and/or insomnia (yes/no) as possible confounding factors [5, 19, 33]. In detail, each regression model was first fitted including one of the sleep and circadian parameters as the dependent variable and both a 2-level group variable (BS patients vs. HCs) and the confounding factors as independent predictors. Secondly, regression models were fitted by replacing the two-level group variable with a three-level group variable dividing participants into the three aforementioned sub-cohorts, considering HCs as the reference level. Treatment for mood disorders and/or insomnia was included only in the latter models to avoid multicollinearity [34].

Moreover, possible associations between either active disease or fibromyalgia and sleep and circadian parameters were explored through *post hoc* non-parametric tests. To confirm the results from the aforementioned tests, we performed *post hoc* multiple linear regression models including one of the sleep and circadian parameters as the dependent variable and the following independent predictors: active disease (active BS patients vs. non-active BS patients and HCs), fibromyalgia (fibromyalgic BS patients vs. non-fibromyalgic patients and HCs) and possible cofounding factors.

Additionally, *post hoc* Pearson’s correlation tests between BDCAF scores and sleep and circadian parameters were performed to explore possible quantitative relationship between disease activity and sleep and circadian parameters (see Supplementary Data S2, available at *Rheumatology* online, for a detailed description of the statistical process). Finally, possible differences in GC exposure variables and fibromyalgia prevalence between different OSAS risk categories were explored through *post hoc* non-parametric tests [5, 35, 36]. The significance level was set at *P* < 0.05.

## Results

### Sociodemographic and clinical data

Forty-five BS patients and 61 age-, BMI-, sex- and smoking habits-matched HCs were enrolled in the study (Table 1). Detailed BS patients’ clinical data and history of organ involvement are reported in Supplementary Table S1, available at *Rheumatology* online. Twelve BS patients were classified as active (27.0%), 23 as fibromyalgic (51.1%), 29 reported either one or both conditions (64.4%).

**Table 1.** Differences in sociodemographic data and sleep and circadian parameters between healthy controls and Behcet’s syndrome patients

Variable^a^	Behcet patients^b^ *N* = 45	Healthy controls^b^ *N* = 61	*P*
Age	48 (42, 54)	49 (36, 58)	>0.9^c^
BMI, kg/m^2^	23.9 (22.2, 27.6)	23.8 (22.4, 27.1)	>0.9^c^
Sex (Females)	30 (67%)	36 (59%)	0.5^d^
Smokers	4 (8.9%)	11 (18%)	0.3^d^
Smoking intensity	0 (0, 0)	0 (0, 0)	0.2^c^
PSQI	8 (6, 11)	5 (3, 7)	**<0.001** ^c^
Poor sleepers	34 (76%)	22 (36%)	**<0.001** ^d^
TST	6.87 (6.30, 7.54)	7.18 (6.44, 7.70)	0.3^c^
SE	92.4 (88.2, 94.8)	94.0 (90.6, 96.1)	**0.033** ^c^
WASO	34 (24, 55)	27 (20, 44)	0.11^c^
SRI	77 (72, 83)	81 (70, 84)	0.7^c^
rMEQ	17 (13, 18)	16 (15, 19)	0.5^c^
Acrophase	15.48 (14.82, 16.03)	15.53 (14.90, 16.17)	0.8^c^
Mid-sleep point	3.10 (2.71, 3.66)	3.18 (2.88, 3.68)	0.7^c^
MESOR	0.63 (0.61, 0.66)	0.60 (0.57, 0.66)	0.091^c^
Amplitude	0.42 (0.39, 0.46)	0.43 (0.40, 0.44)	>0.9^c^
IS	0.86 (0.81, 0.90)	0.88 (0.80, 0.91)	0.6^c^
IV	0.30 (0.26, 0.35)	0.30 (0.26, 0.35)	0.5^c^
RA	0.80 (0.71, 0.88)	0.84 (0.78, 0.89)	0.2^c^
OSAS			**0.003** ^d^
Low	5 (11%)	24 (39%)	
Mild	29 (64%)	22 (36%)	
Moderate	10 (22%)	12 (20%)	
High	1 (2.2%)	3 (4.9%)	

Bold text has been used to highlight *P*-values <0.05.

a PSQI: Pittsburgh Sleep Quality Index; Poor sleeper: PSQI > 5; TST: total sleep time (h); WASO: wake after sleep onset (min); SE: sleep efficiency (%); SRI: sleep regularity index (%); rMEQ: reduced Morningness–Eveningness questionnaire; IS: interdaily stability; IV: interaily variability; RA: relative amplitude; MESOR: midline-estimating statistic of rhythm; OSAS: obstructive sleep apnoea syndrome.

b Median (*Q*1, *Q*3); *n* (%).

c Wilcoxon rank sum test.

d Fisher’s exact test.

### Associations between BS cohort and sleep and circadian parameters

Participants’ sleep and circadian parameters are reported in Table 1. Our cohort of BS patients showed a median PSQI score of 8 (IQR = 6.0–11.0). All 23 patients with comorbid fibromyalgia and 10 of the 12 active BS patients (83%) were classified as poor sleepers (PSQI > 5; *N* = 34, 76%). In parallel, 50% of non-fibromyalgic BS patients (*N* = 11) and 36% of HCs met this criterion (PSQI > 5), with a median PSQI of 5.5 (4.0, 8.0) and 5 (IQR = 3–7), respectively.

**Figure 1.**
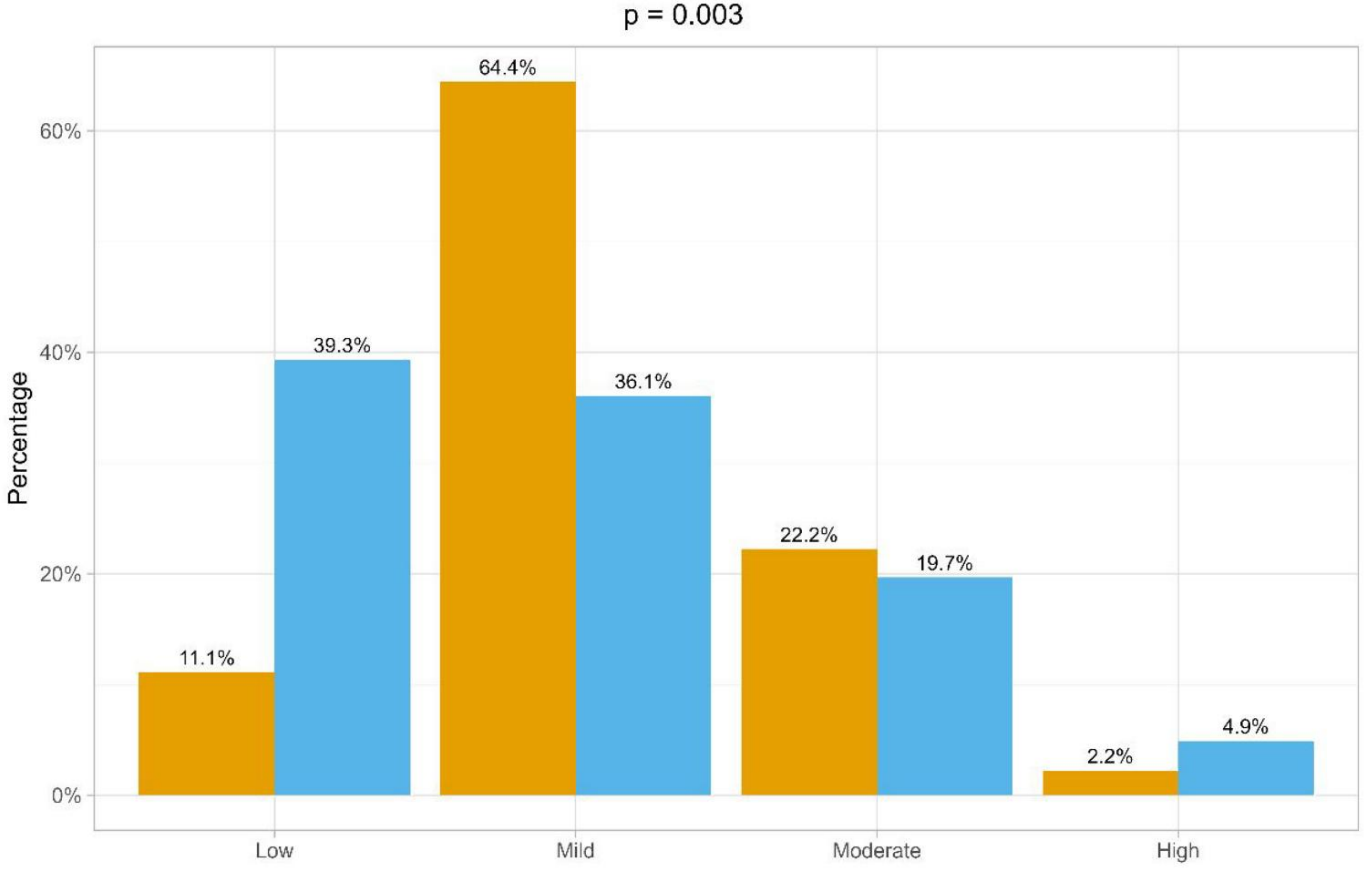
Differences in actigraphic pre-test OSAS risk between Behçet syndrome (BS) patients and healthy controls (HCs). Within each OSAS risk category (i.e., Low, Mild, Moderate, High), the left column represents BS patients, the right column HCs. OSAS: obstructive sleep apnoea syndrome; *P: P*-value (Fisher’s test)

Compared to HCs, BS patients showed lower actigraphic SE (*P* = 0.033) and lower perceived sleep quality, as measured by the PSQI (*P* < 0.001; Supplementary Fig. S1, available at *Rheumatology* online). Furthermore, a different distribution of OSAS risk categories was observed, with BS patients showing a higher risk of OSAS as compared to HCs (*P* = 0.003; Fig. 1). In detail, BS patients were more frequently classified as at moderate risk of OSAS compared to HCs (64% and 36%, respectively). In parallel, BS patients were less frequently classified as at low risk of OSAS compared to HCs (11% and 39%, respectively).

Secondly, participants were divided into three sub-cohorts (i.e. HCs, active and/or fibromyalgic BS patients, and non-active, non-fibromyalgic BS patients), and possible differences in sleep and circadian parameters between the three sub-cohorts were tested (Table 2; Supplementary Fig. S2, available at *Rheumatology* online). Significant differences in PSQI (*P* < 0.001), SE (*P* < 0.001) and WASO (*P* < 0.001) were found between the three groups, with *post hoc* tests showing significantly higher PSQI and sleep fragmentation in active and/or fibromyalgic patients compared to the other two sub-cohorts. Furthermore, lower TST was found in active and/or fibromyalgic BS patients (median = 6.43 h; IQR = 6.13–7.36) compared to non-active, non-fibromyalgic BS patients (median = 7.14 h; IQR = 6.92–7.73; *P* = 0.021) and HCs (median = 7.18; IQR = 6.44–7.70; *P* = 0.072).

**Table 2.** Differences in sociodemographic data and sleep and circadian parameters between participants sub-cohorts

Variable^a^	Healthy controls^A^ *N* = 61^b^	Non-active, non-FM Behcet Patients^B^ *N* = 16^b^	Active and/or FM Behcet Patients^C^ *N* = 29^b^	*P*	*Post hoc* tests		
					*P* ^A^ ^,C^	*P* ^B^ ^,C^	*P* ^A^ ^,B^
Age	49 (36, 58)	50 (43, 54)	48 (42, 54)	>0.9^c^	>0.9^e^	0.9^e^	>0.9^e^
BMI, kg/m^2^	23.8 (22.4, 27.1)	23.5 (22.1, 27.1)	23.9 (22.5, 28.6)	0.9^c^	0.8^e^	0.7^e^	0.8^e^
Sex (females)	36 (59%)	10 (63%)	20 (69%)	0.7^d^	0.4^d^	0.7^d^	0.8^d^
Smokers	11 (18%)	0 (0%)	4 (14%)	0.2^d^	0.8^d^	0.3^d^	0.11^d^
Smoking intensity	0 (0, 0)	0 (0, 0)	0 (0, 0)	0.2^c^	0.6^e^	0.13^e^	0.071^e^
PSQI	5 (3, 7)	5 (4, 8)	10 (8, 13)	**<0.001** ^c^	**<0.001** ^e^	**<0.001** ^e^	0.8^e^
Poor Sleepers	22 (36%)	7 (44%)	27 (93%)	**<0.001** ^d^	**<0.001** ^d^	**<0.001** ^d^	0.6^d^
TST	7.18 (6.44, 7.70)	7.14 (6.92, 7.73)	6.43 (6.13, 7.36)	0.070^c^	0.072^e^	**0.021** ^e^	0.5^e^
SE	94.0 (90.6, 96.1)	95.0 (93.4, 97.0)	90.1 (85.3, 92.4)	**<0.001** ^c^	**<0.001** ^e^	**<0.001** ^e^	0.15^e^
WASO	27 (20, 44)	21 (15, 28)	42 (33, 73)	**<0.001** ^c^	**<0.001** ^e^	**<0.001** ^e^	0.054^e^
SRI	81 (70, 84)	77 (71, 85)	77 (72, 83)	0.7^c^	0.4^e^	0.6^e^	0.8^e^
rMEQ	16 (15, 19)	18 (17, 19)	15 (13, 18)	0.051^c^	0.067^e^	**0.040** ^e^	0.2^e^
Acrophase	15.53(14.90,16.17)	15.55 (14.77, 16.18)	15.48 (14.82, 16.02)	>0.9^c^	0.8^e^	>0.9^e^	0.8^e^
Mid-sleep point	3.18 (2.88, 3.68)	3.21 (2.71, 3.90)	3.10 (2.77, 3.56)	0.8^c^	0.6^e^	0.6^e^	>0.9^e^
MESOR	0.60 (0.57, 0.66)	0.62 (0.58, 0.65)	0.63 (0.61, 0.67)	0.070^c^	**0.026** ^e^	0.11^e^	>0.9^e^
Amplitude	0.43 (0.40, 0.44)	0.42 (0.40, 0.46)	0.43 (0.38, 0.46)	>0.9^c^	0.9^e^	>0.9^e^	0.8^e^
IS	0.88 (0.80, 0.91)	0.83 (0.78, 0.86)	0.87 (0.83, 0.90)	0.12^c^	0.7^e^	**0.022** ^e^	0.10^e^
IV	0.30 (0.26, 0.35)	0.30 (0.27, 0.37)	0.29 (0.26, 0.33)	0.4^c^	0.2^e^	0.2^e^	0.6^e^
RA	0.84 (0.78, 0.89)	0.85 (0.76, 0.90)	0.80 (0.69, 0.86)	0.2^c^	0.089^e^	0.4^e^	>0.9^e^
OSAS risk				**0.004** ^d^	0.069^d^	0.088^d^	**0.004** ^d^
Low	24 (39%)	1 (6.3%)	4 (14%)				
Mild	22 (36%)	14 (88%)	15 (52%)				
Moderate	12 (20%)	1 (6.3%)	9 (31%)				
High	3 (4.9%)	0 (0%)	1 (3.4%)				

For post-hoc tests, participants sub-cohorts are specified by footnotes A (Healthy Controls), B (Non-active, non-FM Behcet Patients), and C (Active and/or FM Behcet Patients).

Bold text has been used to highlight *P*-values <0.05.

a BS: Behçet syndrome; PSQI: Pittsburgh Sleep Quality Index; Poor sleeper: PSQI > 5; TST: total sleep time (h); WASO: wake after sleep onset (min); SE: sleep efficiency (%); SRI: sleep regularity index (%); rMEQ: reduced Morningness–Eveningness questionnaire; IS: interdaily stability; IV: interaily variability; RA: relative amplitude; MESOR: midline-estimating statistic of rhythm.

b Median (*Q*1, *Q*3); *n* (%).

c Kruskal–Wallis rank sum test.

d Fisher’s exact test.

e Wilcoxon rank sum test.

Concerning circadian parameters, a lower rMEQ score was observed in active and/or fibromyalgic BS patients (median = 15; IQR = 13–18) compared to non-active, non-fibromyalgic BS patients (median = 18; IQR = 17–19; *P* = 0.040) and HCs (median = 16; IQR = 15–19; *P* = 0.067), with a tendence towards significance being observed when testing possible differences in rMEQ between the three sub-cohorts (*P* = 0.051). Furthermore, a significantly higher MESOR was found in active and/or fibromyalgic BS patients (median = 0.63; IQR = 0.61–0.67) compared to HCs (median = 0.60; IQR = 0.57–0.66; *P* = 0.026). Finally, active and/or fibromyalgic BS patients showed a significantly higher IS (median = 0.87; IQR = 0.83–0.90) compared to non-active, non-fibromyalgic BS patients (median = 0.83; IQR = 0.78, 0.86; *P* = 0.022). Notably, non-active, non-fibromyalgic BS patients and HCs did not significantly differ in sleep and circadian parameters, except for OSAS risk (*P* = 0.004; Table 2).

*Post hoc* tests showed significant differences in PSQI (*P* < 0.001), SE (*P* = 0.005), WASO (*P* = 0.006) and OSAS risk (*P* = 0.015) between fibromyalgic BS patients, non-fibromyalgic BS patients and HCs (Supplementary Table S2, available at *Rheumatology* online). Similar results were found when testing possible differences in sleep and circadian parameters between active BS patients, non-active BS patients and HCs (Supplementary Table S3, available at *Rheumatology* online). These results were confirmed by *post hoc* multiple linear regressions (Supplementary Table S4, available at *Rheumatology* online), which also showed fibromyalgia to be a significant predictor of TST (*β* = −0.72, *P* = 0.043).

Quantitative analyses showed that for every 1-unit increase in BDCAF score, an average increase of 3.3 points in PSQI score was observed, along with an average decrease of 2.9% in SE, and an average decrease of 4.2% in SRI (Supplementary Data S2, available at *Rheumatology* online). Higher exposure to GC and was observed in participants at mild, and moderate-to-high OSAS risk compared to participants at low OSAS risk (Supplementary Table S5, available at *Rheumatology* online). A higher prevalence of fibromyalgia was observed in participants at mild OSAS risk compared to participants at low OSAS risk (Supplementary Table S5, available at *Rheumatology* online).

### Linear regression models investigating possible associations between BS patients and sleep and circadian parameters

Multiple linear regression models were used to confirm the results from the non-parametric tests, including age, BMI, sex and smoking habit in the models as possible confounding factors.

First, regression models were fitted including one of the sleep and circadian parameters as the dependent variable and both a two-level group variable (BS patients vs. HCs) and the confounding factors as independent predictors (Table 3). In these models, the significant difference in PSQI (*β* = 3.2; *P* < 0.001) and SE (*β* = 3.0; *P* < 0.001) between BS patients and HCs was confirmed. Moreover, age was significantly associated with sleep regularity (SRI; *β* = 0.23; *P* = 0.006), IS (*β* = 0.001; *P* = 0.037) and rMEQ (*β* = 0.06; *P* = 0.012), with older patients showing more regular rest–activity patterns and a significant shift towards morningness. Finally, smoking habit was significantly associated with rMEQ (*β* = −2.4; *P* = 0.010), with weekly cigarette consumption increasing along with eveningness.

**Table 3.** Linear regression models investigating possible associations between BS patients and sleep and circadian parameters

Independent variables^a^	BS patients	Age	BMI	Sex	Smoker	*R* ^²^	Adj-*R*^²^
PSQI	3.2 (<0.001***)	0.03 (0.15)	0.11 (0.2)	−1.2 (0.062)	1.0 (0.3)	0.253	0.216
TST	−0.22 (0.3)	−0.01 (0.2)	0.01 (0.9)	−0.19 (0.4)	−0.39 (0.2)	0.032	−0.017
SE	−2.3 (0.031*)	−0.01 (0.7)	−0.14 (0.3)	0.23 (0.8)	1.9 (0.2)	0.083	0.037
WASO	8.8 (0.081)	0.03 (0.9)	0.53 (0.4)	−2.5 (0.6)	−9.2 (0.2)	0.064	0.017
SRI	0.96 (0.7)	0.23 (0.006**)	−0.45 (0.10)	−1.3 (0.5)	−0.72 (0.8)	0.116	0.072
rMEQ	−0.94 (0.13)	0.06 (0.012*)	−0.02 (0.8)	−0.86 (0.2)	−2.4 (0.010*)	0.189	0.149
Acrophase	−0.01 (>0.9)	−0.01 (0.12)	0.00 (0.9)	−0.26 (0.2)	0.19 (0.5)	0.057	0.010
Mid-sleep point	−0.01 (>0.9)	−0.01 (0.3)	0.01 (0.6)	−0.28 (0.10)	0.38 (0.13)	0.072	0.026
Amplitude	−0.00 (0.9)	0.00 (0.7)	0.00 (0.8)	0.00 (>0.9)	0.02 (0.13)	0.025	−0.024
MESOR	0.02 (0.089)	0.00 (0.6)	0.00 (0.3)	0.00 (0.8)	0.02 (0.2)	0.056	0.008
IS	−0.01 (0.6)	0.00 (0.037*)	0.00 (0.8)	0.01 (0.6)	−0.01 (0.6)	0.064	0.017
IV	−0.01 (0.3)	0.00 (0.7)	0.00 (0.2)	0.01 (0.6)	−0.03 (0.082)	0.058	0.011
RA	−0.03 (0.12)	0.00 (>0.9)	0.00 (0.3)	0.00 (>0.9)	0.04 (0.3)	0.054	0.006

a BS: Behçet syndrome; smoker (yes/no); PSQI: Pittsburgh Sleep Quality Index; TST: total sleep time (h); WASO: wake after sleep onset (min); SE: sleep efficiency (%); SRI: sleep regularity index (%); IS: interdaily stability; IV: interaily variability; RA: relative amplitude; MESOR: midline-estimating statistic of rhythm; rMEQ: reduced Morningness–Eveningness questionnaire.

Bold text has been used to highlight *P*-values <0.05. *P-value < 0.05, **P-value < 0.01, ***P-value < 0.001.

**Figure 2.**
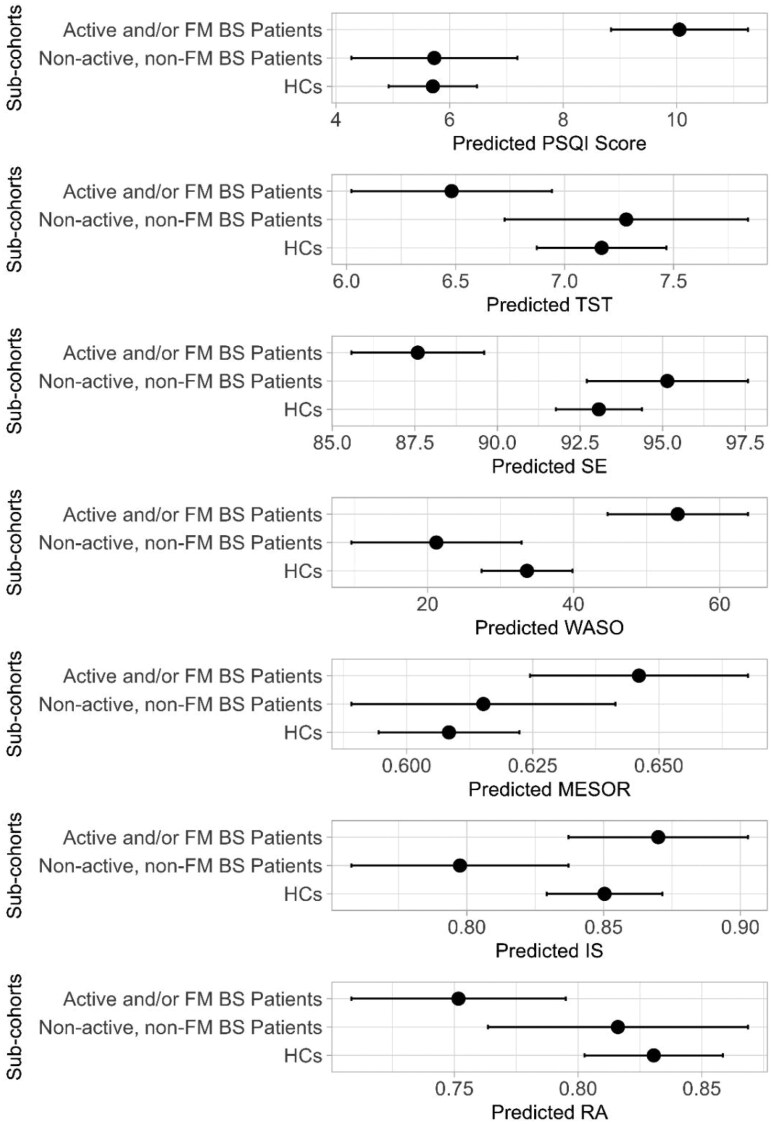
Predicted values of PSQI, TST, SE, WASO, MESOR, IS and RA in participants sub-cohorts. BS: Behçet syndrome; FM: fibromyalgia; PSQI: Pittsburgh Sleep Quality Index; TST: total sleep time; WASO: wake after sleep onset (min); SE: sleep efficiency (%); MESOR: midline-estimating statistic of rhythm; IS: interdaily stability; RA: relative amplitude

Secondly, regression models were fitted by replacing the two-level group variable with a three-level group variable dividing participants into three sub-cohorts, that is HCs (reference level), active and/or fibromyalgic BS patients, and non-active, non-fibromyalgic BS patients (Table 4; Fig. 2). In these models, significant differences in PSQI (*P* < 0.001), SE (*P* < 0.001), WASO (*P* < 0.001), TST (*P* = 0.042), MESOR (*P* = 0.026), RA (*P* = 0.019) and IS (*P* = 0.018) were confirmed between the three sub-cohorts (Table 4), with BS patients with active disease and/or fibromyalgia showing significantly lower predicted values of SE and significantly higher predicted values of PSQI and WASO compared to the other two sub-cohorts (Fig. 2). Moreover, a tendence towards reduction in TST was observed in active and/or fibromyalgic patients compared to the other two sub-cohorts (Fig. 2). In parallel, active and/or fibromyalgic BS patients showed significantly higher predicted values of IS compared to non-active, non-fibromyalgic BS patients and both higher predicted MESOR and lower RA compared to HCs.

**Table 4.** Linear regression models investigating possible associations between participants sub-cohorts and sleep and circadian parameters

	Group variable										
Independent variables^a^	HCs	Active and/or FM BS patients	Non-active, non-FM BS patients	*P*	Age *β* (*P*)	BMI *β* (*P*)	Sex *β* (*P*)	Smoker *β* (*P*)	Mood disorders/insomnia treatment *β* (*P*)	*R* ^²^	Adj-*R*^²^
PSQI	Ref.	4.3	0.03	<0.001***	0.03 (0.2)	0.10 (0.2)	−1.1 (0.076)	0.42 (0.6)	0.74 (0.5)	0.395	0.352
TST	Ref.	−0.69	0.11	0.042*	−0.01 (0.2)	0.00 (>0.9)	−0.18 (0.4)	−0.36 (0.3)	0.71 (0.086)	0.086	0.021
SE	Ref.	−5.5	2.1	<0.001***	−0.01 (0.7)	−0.15 (0.2)	0.12 (>0.9)	2.6 (0.075)	2.1 (0.3)	0.252	0.199
WASO	Ref.	21	−12	<0.001***	0.00 (>0.9)	0.48 (0.4)	−1.6 (0.7)	−13 (0.060)	−1.3 (0.9)	0.224	0.168
SRI	Ref.	−0.32	2.4	0.7	0.23 (0.007**)	−0.45 (0.10)	−1.4 (0.5)	−0.50 (0.9)	1.2 (0.8)	0.122	0.058
rMEQ	Ref.	−1.3	0.53	0.2	0.06 (0.006**)	0.00 (>0.9)	−0.97 (0.12)	−2.1 (**0.026**)	−1.1 (0.3)	0.236	0.181
Acrophase	Ref.	−0.25	−0.01	0.6	−0.01 (0.077)	−0.01 (0.7)	−0.24 (0.2)	0.16 (0.6)	0.58 (0.092)	0.084	0.019
Mid-sleep point	Ref.	−0.26	0.17	0.3	−0.01 (0.3)	0.01 (0.7)	−0.27 (0.11)	0.39 (0.11)	0.40 (0.2)	0.101	0.037
Amplitude	Ref.	−0.01	0	0.7	0.00 (>0.9)	0.00 (>0.9)	0.00 (0.8)	0.02 (0.2)	0.03 (0.049*)	0.063	−0.004
MESOR	Ref.	0.04	0.01	0.026*	0.00 (0.5)	0.00 (0.2)	0.00 (>0.9)	0.02 (0.2)	−0.03 (0.085)	0.098	0.034
IS	Ref.	0.02	−0.05	0.020*	0.001 (0.042*)	0.00 (0.9)	0.01 (0.5)	−0.02 (0.4)	−0.01 (0.8)	0.135	0.073
IV	Ref.	−0.01	0	0.8	0.00 (0.8)	0.00 (0.3)	0.01 (0.7)	−0.03 (0.14)	−0.03 (0.2)	0.083	0.018
RA	Ref.	−0.08	−0.01	0.019*	0.00 (0.9)	0.00 (0.14)	0.00 (0.9)	0.03 (0.3)	0.09 (0.024*)	0.114	0.05

a BS: Behçet Syndrome; FM: fibromyalgic; HCs: healthy controls; PSQI: Pittsburgh Sleep Quality Index; TST: total sleep time (h); WASO: wake after sleep onset (min); SE: sleep efficiency (%); SRI: sleep regularity index (%); IS: interdaily stability; IV: interaily variability; RA: relative amplitude; MESOR: midline-estimating statistic of rhythm; rMEQ: reduced Morningness–Eveningness questionnaire.

Bold text has been used to highlight *P*-values <0.05. *P-value < 0.05, **P-value < 0.01, ***P-value < 0.001.

## Discussion

This study confirms that individuals with BS experience poorer sleep quality and more fragmented sleep compared to healthy individuals. However, our findings demonstrate that such sleep disturbances are primarily linked to active disease and comorbid fibromyalgia, irrespective of factors like age, BMI, sex, smoking habits and treatment for mood disorders and/or insomnia. Specifically, we observed no significant differences in sleep parameters between HCs and BS patients without active disease and comorbid fibromyalgia. Importantly, our research is the first to highlight differences in robustness of circadian rhythms between BS patients with active disease and/or fibromyalgia and HCs. Finally, our results corroborate previous studies that have identified an increased risk of obstructive sleep apnoea syndrome (OSAS) in all BS patients compared to healthy individuals.

Sleep impairment has been reported in up to 73.6% of BS patients [1] and represents one of the primary factors impacting BS patients’ QoL [1, 2], along with active disease and comorbid fibromyalgia [37]. Within this context, our data more clearly define the relationship between active disease, comorbid fibromyalgia and sleep impairment in BS patients. In detail, we previously described different BS patients’ sleep disruption patterns associated with active disease and comorbid fibromyalgia [5]; with the present study, we demonstrated that both active disease and comorbid fibromyalgia are two main correlates of BS patients sleep impairment, with HCs and BS patients without those conditions non-significantly differing in sleep parameters. As a whole, these data may suggest that BS patients’ QoL impairment caused by either active disease or comorbid fibromyalgia may be partially mediated by sleep impairment. However, this hypothesis remains to be tested in future studies. Confirming this hypothesis may encourage greater attention to sleep disturbance screening and management in clinical practice, potentially improving patients’ QoL independent of disease activity and comorbid fibromyalgia.

To the best of our knowledge, this is the first study comparing circadian rhythm parameters between BS patients and HCs, contributing to the growing body of literature investigating circadian rhythm in immune-mediated diseases [13]. More specifically, we found that active and/or fibromyalgic patients show lower RA and higher mean accelerometric activity as measured by MESOR, compared to HCs. These results suggest lower robustness of circadian rhythms in this population, consistent with the observed sleep parameters alterations [38, 39]. Moreover, older participants showed a significant shift towards morningness, while smokers shifted towards eveningness, consistent with previous findings in the general population [27, 33]. As previously reported [5], active and/or fibromyalgic BS patients exhibited a significantly more regular daily rest–activity pattern than non-active, non-fibromyalgic BS patients, which may contribute to pain and fatigue management in fibromyalgic BS patients [3, 40].

Our results confirm a higher OSAS risk in BS patients compared to healthy individuals, irrespective of age, sex, BMI and smoking habits. Moreover, the absence of clinical manifestations that may increase the risk of OSAS in our BS cohort, such as SVCS [17], suggests that BS patients may suffer from an increased OSAS risk irrespective of their clinical manifestations [8, 9]. Nevertheless, such significant increase in OSAS risk may not be severe. In a four-level scale (i.e. low, mild, moderate, high), recruited BS patients were indeed more frequently classified as at mild risk of OSAS (64%), and less frequently classified as at low risk of OSAS (11%) compared to HCs (36% and 39%, respectively), with minor discrepancies in risk categorization across the moderate and the high OSAS risk categories. Moreover, our results suggest that exposure to GC and fibromyalgia may be associated to higher OSAS risk categories in BS patients, in line with previous literature [35, 36]. However, such results should be confirmed through longitudinal studies including wider BS cohorts and investigating possible confounding factors. Finally, future studies should verify the OSAS risk with diagnostic testing,i.e. polysomnography or home sleep apnoea testing.

Sleep disturbances have been observed also in other forms of vasculitis [41]. A recent study found that 77.7–80.7% of ANCA-vasculitis patients report disturbed sleep based on PSQI scores [41]. These results align with our data and suggest the possible presence of a common pathogenesis underlying sleep disturbances in vasculitides, such as the effect of inflammatory cytokines on sleep continuity and propensity, the exposure to GC and the presence of painful disease manifestations [1, 5, 36, 41]. Moreover, the prevalence of comorbid fibromyalgia is remarkably higher in autoimmune diseases—included valculitides—compared to the general population [4, 42], with sleep impairment being a key feature of fibromyalgia [3]. Accordingly, fibromyalgia has been associated to reduced sleep quality, duration and maintenance in rheumatologic diseases [4, 5, 42]. Within this context, a multidimensional sleep characterization including both subjective and objective measures may help distinguishing sleep patterns associated with specific sleep disturbing factors [5, 25, 43].

The observed 51.1% fibromyalgia prevalence in our BS cohort is notably higher than the prevalence reported in previous studies enrolling BS patients (5.7–37.1%) [4] or patients suffering from different vasculitides [41]. This inconsistency may be explained by both discrepancies in fibromyalgia diagnostic criteria and variability in gender composition across studies [4, 44, 45]. The AAPT criteria have been shown to identify fibromyalgia with milder symptoms compared to the 2016 and 1990 ACR criteria [4, 44]. Unlike the 2016 ACR’s generalized pain definition, the AAPT definition of multisite pain indeed allows for the examination of primarily non-musculoskeletal sites (head, chest, abdomen), which has been shown to impact the prevalence of fibromyalgia [44]. In parallel, the 1990 ACR criteria focus exclusively on tender points examination and do not include the investigation of sleep disturbances or fatigue [44], in contrast to the AAPT criteria [23]. Furthermore, prior studies in BS cohorts relied only on consultant diagnosis without specifying fibromyalgia diagnostic criteria, potentially contributing to fibromyalgia prevalence variability [45]. Finally, the variability in gender composition across BS cohorts may partially account for FM prevalence variation across previous studies. Indeed, the reported male-to-female ratios range from 0.36 to 5.4 [46, 47] with fibromyalgia being more frequent among females [4, 42]. These findings, along with the female predominance in our cohort (62%), likely contribute to both the heterogeneity in fibromyalgia prevalence across studies and the observed fibromyalgia prevalence in our BS population.

Furthermore, a relatively low prevalence of arthralgia was observed in our cohort of BS patients with comorbid fibromyalgia at the time of recruitment (13%). This apparent discrepancy with previous literature may depend on the severity of fibromyalgia symptoms at recruitment. The retrospective review of clinical records indeed showed a 48% prevalence of arthralgia throughout the entire clinical history of fibromyalgic BS patients, in line with previous literature [4, 48, 49]. Notably, all fibromyalgia diagnoses predated recruitment, when specific treatment was suggested, potentially contributing to symptoms’ severity reduction.

One of the limitations of our study is that our BS cohort included a relatively limited number of active BS patients (*N* = 12, 26.6%), which may depend on the timing of the recruitment phase (i.e. outpatient visits), when disease activity is generally better controlled in comparison to inpatient settings. Moreover, half of the recruited active BS patients showed comorbid fibromyalgia. Further studies should aim at confirming our results in cohorts of BS patients including a larger number of active BS patients without comorbid fibromyalgia. Finally, a relatively low prevalence of small fiber neuropathy is reported in our cohort of BS patients, possibly depending on sampling bias, and the cross-sectional nature of our study design prevents us to longitudinally explore a possible causal relationship binding fibromyalgia, active disease, sleep and circadian rhythm disturbances.

This study confirms that BS patients show lower sleep quality and higher sleep fragmentation compared to HCs and demonstrates that such sleep impairment is primarily related to active disease and comorbid fibromyalgia, irrespective of age, BMI, sex or smoking habits. Moreover, we identified for the first time significant differences in robustness of circadian rhythms between BS patients and HCs. Finally, we found a significantly—though not severely—higher pre-test OSAS risk in BS patients compared to HCs. Our data support incorporating screening tests to investigate BS patients’ sleep and circadian rhythm disturbances into clinical practice, especially in BS patients suffering from active disease or comorbid fibromyalgia.

## Supplementary Material

keaf326_Supplementary_Data

## Data Availability

The data underlying this article will be shared on reasonable request to the corresponding author.
