# Supplementary material for: Sleep and circadian parameters in Behçet’s syndrome: a comparative analysis using actigraphy and questionnaires
Source: Rheumatology (Oxford). 2025 Jun 9;64(10):5460–70. doi: 10.1093/rheumatology/keaf326 (PMC12494212; doi:10.1093/rheumatology/keaf326)
Supplement: keaf326_Supplementary_Data [file keaf326_supplementary_data.zip › keaf326_Supplementary_Data/rhe-25-0841-File002.docx]

# Supplementary Data

## Supplementary Data S1

The following accelerometric sleep parameters were estimated in the present study:

- Total Sleep Time (TST): the total duration of sleep (hours). It is defined as the sum of all asleep time within a sleep period;
- Waking After Sleep Onset (WASO): the time spent awake within a sleep period (minutes);
- Sleep Efficiency (SE): the time spent asleep within a sleep period, expressed as a percentage of the sleep period (%). WASO and SE were considered as measures of sleep fragmentation (1,2);
- Sleep Regularity Index (SRI): the likelihood of the same sleep-wake state occurring in epochs that are 24 hours apart (%). SRI measures the similarity of sleep-wake patterns between consecutive days (1,2);
- Mid-sleep point: the middle of the sleep period between the sleep onset and ﬁnal awakening, calculated by adding to the average sleep onset half of the average total sleep time (average sleep onset + average TST/2) (1,2).

The following accelerometric circadian parameters were estimated in the present study:

- Midline-estimating statistic of rhythm (MESOR): a rhythm-adjusted mean. It represents the mean of the activity level as modelled by the sine wave;
- Amplitude: half of the peak-to-nadir difference, a measure of the extent of predictable variation within a cycle. More robust rhythms have a higher amplitude;
- Acrophase: timing of peak activity or the point in the cycle with highest activity;
- Interdaily stability (IS): estimates the variability in rest–activity patterns across all days. It is a measure of rest–activity rhythms regularity. It is expressed as values ranging from 0 to 1. Higher values indicate greater stability between days;
- Intradaily variability (IV): quantiﬁes the fragmentation and magnitude of rest–activity transitions within each day. It is usually expressed as values ranging from 0 to approximately 2. Higher values indicate frequent transitions between rest and activity (i.e., frequent naps, increased night-time awakenings);
- Relative amplitude: measures the robustness of the 24 h rest–activity rhythm by calculating the normalized mean difference in activity between the most active 10 h and the least active 5 h, ranging from 0 to 1. Higher values indicate lower activity during the night and high activity during the day, i.e., increased robustness of rest–activity rhythm.

## Supplementary Data S2

To explore possible quantitative relationship between disease activity and sleep and circadian parameters, post-hoc Pearson’s correlation tests between BDCAF score and sleep and circadian parameters were performed. Due to the relatively low number of active BS patients in the recruited BS patients’ group, the distribution of BDCAF data was examined prior to statistical testing to assess potential deviations from normality (see the density plot of BDCAF below) (3). A Shapiro-Wilk test confirmed the non-normal distribution of BDCAF scores (p<0.001).


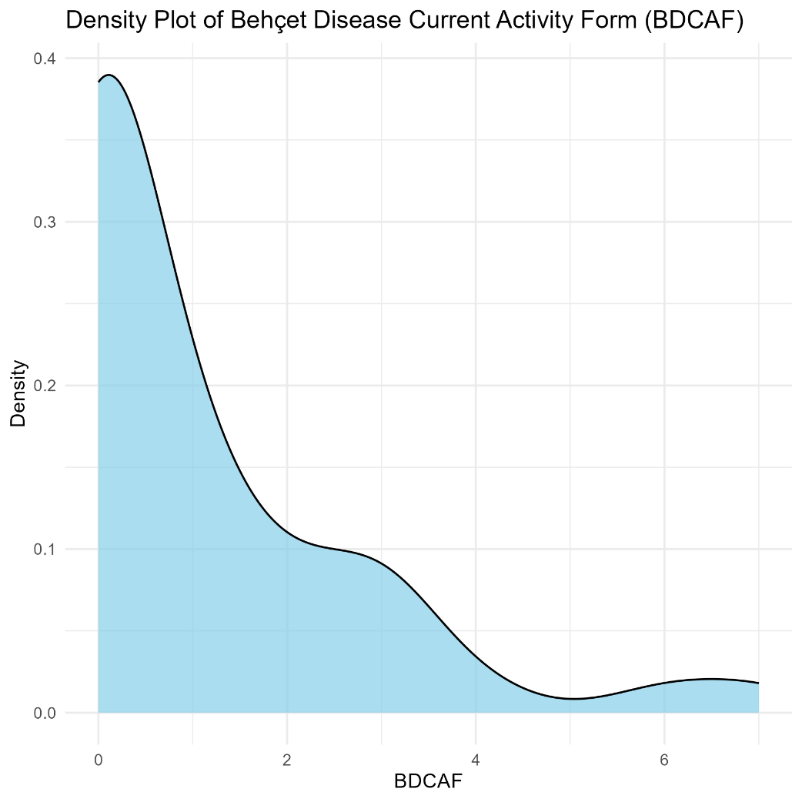


Because of the non-normal distribution of BDCAF data, we first aimed at normalizing BDCAF distribution by applying a logarithmic transformation to non-zero BDCAF values (3) and subsequently conducted Pearson's correlation tests between transformed BDCAF scores and sleep and circadian parameters within the BS patients’ group. Statistically significant correlations between the logarithm of BDCAF and PSQI (β = 0.33, p = 0.028), SE (β= − 0.29, p = 0.049), and SRI (β = -0.42, p=0.004) were found. These findings suggest that for every 1-unit increase in BDCAF score (corresponding to a 10-fold increase in the logarithm of BDCAF), an average increase of 3.3 points in PSQI score was observed, along with an average decrease of 2.9% in SE and an average decrease of 4.2% in SRI.

## References quoted in Supplementary Data

1. American National Standards Institute (ANSI), Consumer Technology Association (CTA), National Sleep Fundation (NSA). Definitions and Characteristics for Wearable Sleep Monitors (ANSI/CTA/NSF-2052.1-A) [Internet]. Arlington, VA, USA: Consumer Technology Association (CTA); 2022 [cited 2023 Sep 29]. Available from: https://shop.cta.tech/products/definitions-and-characteristics-for-wearable-sleep-monitors-ansi-cta-nsf-2052-1-a

2. Smith MT, McCrae CS, Cheung J, Martin JL, Harrod CG, Heald JL, et al. Use of Actigraphy for the Evaluation of Sleep Disorders and Circadian Rhythm Sleep-Wake Disorders: An American Academy of Sleep Medicine Clinical Practice Guideline. Journal of Clinical Sleep Medicine [Internet]. 2018 Jul 15 [cited 2022 Dec 10];14(7):1231–7. Available from: https://jcsm.aasm.org/doi/10.5664/jcsm.7230

3. Rasmussen JL, Dunlap WP. Dealing with Nonnormal Data: Parametric Analysis of Transformed Data vs Nonparametric Analysis. Educ Psychol Meas [Internet]. 1991 Dec [cited 2025 May 1];51(4):809–20. Available from: https://journals.sagepub.com/doi/10.1177/001316449105100402
