# Supplementary material for: Sleep and circadian parameters in Behçet’s syndrome: a comparative analysis using actigraphy and questionnaires
Source: Rheumatology (Oxford). 2025 Jun 9;64(10):5460–70. doi: 10.1093/rheumatology/keaf326 (PMC12494212; doi:10.1093/rheumatology/keaf326)

# Supplementary Data

| **SUPPLEMENTARY TABLE S1: BS patients’ Sociodemographic and Clinical Data** | | | | | | | | |
| --- | --- | --- | --- | --- | --- | --- | --- | --- |
|  | **BS Patients** | **Active Disease** | | | | **Fibromyalgia** | | |
| **Clinical Data**^1^ | **N =** 45^2^ | **No**,  N = 33^2^ | **Yes**,  N = 12^2^ | **p** | | **No**,  N = 22^2^ | **Yes**,  N = 23^2^ | **p** |
| Age | 48 (42, 54) | 50 (45, 62) | 45 (40, 48) | **0.036***^3^* | | 48 (44, 51) | 48 (42, 59) | 0.7*^3^* |
| BMI | 23.9 (22.2, 27.6) | 24.2 (22.2, 27.8) | 23.7 (22.4, 25.9) | 0.9*^3^* | | 24.1 (22.4, 26.5) | 23.9 (21.4, 29.6) | 0.8*^3^* |
| Sex |  |  |  | 0.2*^4^* | |  |  | 0.12*^4^* |
| *Female* | 30 (67%) | 24 (73%) | 6 (50%) |  | | 12 (55%) | 18 (78%) |  |
| *Male* | 15 (33%) | 9 (27%) | 6 (50%) |  | | 10 (45%) | 5 (22%) |  |
| Disease Duration | 5 (4, 13) | 6 (4, 13) | 4 (3, 12) | 0.4^3^ | | 6 (3, 13) | 5 (4, 12) | 0.6 |
| BDCAF | 0 (0, 2) | 0 (0, 0) | 3 (2, 3) | **<0.001^3^** | | 0 (0, 2) | 0 (0, 2) | 0.7 |
| Active Disease | 12 (27%) |  |  |  | | 6 (27%) | 6 (26%) | >0.9^4^ |
| **Clinical Manifestations at the time of recruitment** | | | | | | | | |
| Mouth ulcers | 13 (29%) | 3 (9.1%) | 10 (83%) | **<0.001^4^** | | 5 (23%) | 8 (35%) | 0.5 |
| Genital ulcers | 3 (6.7%) | 0 (0%) | 3 (25%) | **0.016^4^** | | 2 (9.1%) | 1 (4.3%) | 0.6^4^ |
| Erythema nodosum | 3 (6.7%) | 0 (0%) | 3 (25%) | **0.016^4^** | | 2 (9.1%) | 1 (4.3%) | 0.6 |
| Pseudofolliculitis | 5 (11%) | 0 (0%) | 5 (42%) | **<0.001^4^** | | 3 (14%) | 2 (8.7%) | 0.7^4^ |
| Arthralgia | 8 (18%) | 2 (6.1%) | 6 (50%) | **0.002^4^** | | 5 (23%) | 3 (13%) | 0.5 |
| Arthritis | 2 (4.4%) | 0 (0%) | 2 (17%) | 0.067^4^ | | 1 (4.5%) | 1 (4.3%) | >0.9^4^ |
| Uveitis | 2 (4.4%) | 1 (3%) | 1 (8.3%) | 0.5^4^ | | 1 (4.5%) | 1 (4.3%) | >0.9 |
| Headache | 1 (2.2%) | 0 (0%) | 1 (8.3%) | 0.3^4^ | | 0 (0%) | 1 (4.3%) | >0.9^4^ |
| Diarrhea | 5 (11%) | 0 (0%) | 5 (45%) | **0.001**^4^ | | 1 (4.8%) | 4 (17%) | 0.3^4^ |
| Superficial vein thrombosis | 1 (2.2%) | 1 (2.7%) | 0 (0%) | >0.9^4^ | | 0 (0%) | 1 (4.3%) | >0.9^4^ |
| Fibromyalgia | 23 (51%) | 17 (52%) | 6 (50%) | >0.9^4^ | |  |  |  |
| HLAB*51 mutation (N=30) | 18 (60%) | 14 (61%) | 4 (57%) | >0.9^4^ | | 8 (57%) | 10 (63%) | >0.9^4^ |
| *Not tested* | 15 (33%) | 9 (27%) | 5 (41%) |  | | 8 (36%) | 7 (30%) |  |
| **Immunomodulatory and immunosuppressive treatment** | | | | | | | | |
| Adalimumab | 10 (23%) | 7 (23%) | 3 (25%) |  | | 5 (23%) | 5 (24%) |  |
| Azathioprine | 9 (21%) | 7 (23%) | 2 (17%) |  | | 4 (18%) | 5 (24%) |  |
| Canakinumab | 1 (2.3%) | 0 (0%) | 1 (8.3%) |  | | 1 (4.5%) | 0 (0%) |  |
| Colchicine | 14 (33%) | 10 (32%) | 4 (33%) |  | | 6 (27%) | 8 (38%) |  |
| Cyclosporine | 1 (2.3%) | 1 (3.2%) | 0 (0%) |  | | 1 (4.5%) | 0 (0%) |  |
| Infliximab | 2 (4.7%) | 0 (0%) | 2 (17%) |  | | 1 (4.5%) | 1 (4.8%) |  |
| Methotrexate | 1 (2.3%) | 1 (3.2%) | 0 (0%) |  | | 0 (0%) | 1 (4.8%) |  |
| None | 5 (12%) | 5 (16%) | 0 (0%) |  | | 4 (18%) | 1 (4.8%) |  |
| Biologic Treatment | 34 (76%) | 24 (73%) | 10 (83%) | >0.9 | | 18 (82%) | 16 (70%) | 0.5^4^ |
| **Glucocorticoids Exposure** | | | | | | | | |
| GC Treatment | 18 (40%) | 14 (42%) | 4 (33%) | 0.7^4^ | | 6 (27%) | 12 (52%) | 0.13 |
| dGC | 5.0 (5.0, 8.8) | 5.0 (5.0, 5.0) | 12.5 (5, 20.0) | 0.15^4^ | | 5.0 (5.0, 8.8) | 5.0 (5.0, 6.9) | 0.7^3^ |
| One-year cumulative GC dose | 104 (0, 1,400) | 168 (0, 1,460) | 42 (0, 844) | 0.5^3^ | | 42 (0, 345) | 449 (0, 1,460) | 0.3^3^ |
|  |  |  |  | |  |  |  |  |
|  |  |  |  | |  |  |  |  |
| **Mood Disorders and Insomnia Treatment** | | | | | | | | |
| Mood Disorders and/or Insomnia Treatment | 12 (27%) | 10 (30%) | 2 (17%) | | 0.5*^4^* | 1 (4.5%) | 11 (48%) | **0.002***^4^* |
| *Trazodone* | 3 (6.7%) | 3 (9.1%) | 0 (0%) | |  | 1 (4.5%) | 2 (8.7%) |  |
| *Zolpidem* | 1 (2.2%) | 0 (0%) | 1 (8.3%) | |  | 0 (0%) | 1 (4.3%) |  |
| *SSRIs* | 5 (11%) | 4 (12%) | 1 (8.3%) | |  | 1 (4.5%) | 4 (17%) |  |
| *SNRIs* | 1 (2.2%) | 1 (3.0%) | 0 (0%) | |  | 0 (0%) | 1 (4.3%) |  |
| *TCAs* | 2 (4.4%) | 1 (3.0%) | 1 (8.3%) | |  | 0 (0%) | 2 (8.7%) |  |
| *Mood stabilizers* | 3 (6.7%) | 3 (9.1%) | 0 (0%) | |  | 0 (0%) | 3 (13%) |  |
| *Gabapentinoids* | 4 (8.9%) | 3 (9.1%) | 1 (8.3%) | |  | 0 (0%) | 4 (17%) |  |
| **History of organ involvement** | | | | | | | | |
| Mouth ulcers | 41 (91%) | 30 (91%) | 11 (92%) | | >0.9^4^ | 20 (91%) | 21 (91%) | >0.9^4^ |
| Genital ulcers | 24 (53%) | 16 (48%) | 8 (67%) | | 0.3^4^ | 13 (59%) | 11 (48%) | 0.6 |
| Erythema nodosum | 8 (18%) | 6 (18%) | 2 (17%) | | >0.9^4^ | 5 (23%) | 3 (13%) | 0.5^4^ |
| Pseudofolliculitis | 12 (27%) | 5 (15%) | 7 (58%) | | **0.007**^4^ | 6 (27%) | 6 (26%) | >0.9 |
| Arthralgia | 17 (38%) | 10 (30%) | 7 (58%) | | 0.2^4^ | 6 (27%) | 11 (48%) | 0.2^4^ |
| Arthritis | 10 (22%) | 5 (15%) | 5 (42%) | | 0.1^4^ | 3 (14%) | 7 (30%) | 0.3^4^ |
| Ocular involvement |  |  |  | | 0.3^4^ |  |  | 0.4^4^ |
| *Anterior Uveitis* | 1 (2.2%) | 1 (3.0%) | 0 (0%) | |  | 1 (4.5%) | 0 (0%) |  |
| *Episcleritis* | 2 (4.4%) | 1 (3.0%) | 1 (8.3%) | |  | 2 (9.1%) | 0 (0%) |  |
| *Panuveitis* | 16 (36%) | 13 (39%) | 3 (25%) | |  | 6 (27%) | 10 (43%) |  |
| *Posterior Uveitis* | 3 (6.7%) | 1 (3.0%) | 2 (17%) | |  | 2 (9.1%) | 1 (4.3%) |  |
| Stroke | 3 (6.8%) | 2 (6.3%) | 1 (8.3%) | | >0.9^4^ | 0 (0%) | 3 (13%) | 0.2^4^ |
| Small fiber neuropathy | 1 (2.2%) | 0 (0%) | 1 (8.3%) | | >0.9^4^ | 0 (0%) | 1 (4.3%) | >0.9^4^ |
| Diarrhea | 15 (33%) | 9 (27%) | 6 (50%) | | 0.2^4^ | 5 (23%) | 10 (43%) | 0.2^4^ |
| Endoscopic GI features |  |  |  | | 0.3^4^ |  |  | 0.6^4^ |
| *Aphtous colorectal lesions* | 1 (2.2%) | 1 (3.0%) | 0 (0%) | |  | 1 (4.5%) | 0 (0%) |  |
| *IBD-like colorectal lesions* | 2 (4.4%) | 1 (3.0%) | 1 (8.3%) | |  | 1 (4.5%) | 1 (4.3%) |  |
| *Terminal Ileitis* | 1 (2.2%) | 0 (0%) | 1 (8.3%) | |  | 1 (4.5%) | 0 (0%) |  |
| Superficial vein thrombosis | 4 (8.9%) | 2 (6.1%) | 2 (17%) | | 0.3^4^ | 2 (9.1%) | 2 (8.7%) | >0.9^4^ |
| Deep vein thrombosis | 3 (6.7%) | 1 (3.0%) | 2 (17%) | | 0.2^4^ | 3 (14%) | 0 (0%) | 0.11^4^ |
| Modified from Colitta et al (2025) ^1^ BS: Behçet Syndrome; BDCAF: Behçet Disease Current Activity Form - Interval Scale; Active Disease: BDCAF ≥ 2; HLAB*51: Human Leukocyte Antigens B51 (not tested in 15 patients); Biologic Treatment: BS patients treated with biologic agents; dGC: daily glucocorticoid dose (prednisone equivalents; mg). One-year cumulative GC dose: the total amount of GCs administered to a patient over the previous year (prednisone equivalents; mg). | | | | | | | | |
| ^2^Median (IQR); n (%) | | | | | | | | |
| ^3^Wilcoxon rank sum test | | | | | | | | |
| ^4^Fisher's exact test | | | | | | | | |

| **SUPPLEMENTARY TABLE S2 Differences in sociodemographic data and sleep and circadian parameters between fibromyalgic (FM) BS patients, non-FM BS patients and healthy controls (HCs)** | | | | |
| --- | --- | --- | --- | --- |
| **Variable***^1^* | **FM BS patients** N = 23*^2^* | **Non-FM BS patients** N = 22*^2^* | **HCs** N = 61*^2^* | **p** |
| Age | 48 (42, 62) | 48 (44, 51) | 49 (36, 58) | 0.8*^3^* |
| BMI | 23.9 (20.7, 30.7) | 24.1 (22.3, 26.5) | 23.8 (22.4, 27.1) | >0.9*^3^* |
| Sex (Female) | 18 (78%) | 12 (55%) | 36 (59%) | 0.2*^4^* |
| Smokers | 4 (17%) | 0 (0%) | 11 (18%) | 0.081*^5^* |
| Smoking intensity | 0 (0, 0) | 0 (0, 0) | 0 (0, 0) | 0.11*^3^* |
| PSQI | 10.0 (8.0, 13.0) | 5.5 (4.0, 8.0) | 5.0 (3.0, 7.0) | **<0.001***^3^* |
| Poor Sleepers | 23 (100%) | 11 (50%) | 22 (36%) | **<0.001***^4^* |
| TST | 6.60 (6.13, 7.54) | 7.04 (6.49, 7.68) | 7.18 (6.44, 7.70) | 0.3*^3^* |
| SE | 90.9 (80.8, 94.1) | 94.2 (90.1, 95.9) | 94.0 (90.6, 96.1) | **0.005***^3^* |
| WASO | 37 (29, 79) | 25 (17, 40) | 27 (20, 44) | **0.006***^3^* |
| SRI | 77 (72, 83) | 77 (70, 85) | 81 (70, 84) | 0.9*^3^* |
| rMEQ | 16 (13, 18) | 17 (14, 19) | 16 (15, 19) | 0.5*^3^* |
| Acrophase | 15.55 (15.20, 16.03) | 15.21 (14.62, 16.02) | 15.53 (14.90, 16.17) | 0.5*^3^* |
| Mid-sleep point | 3.27 (2.89, 3.64) | 3.02 (2.49, 3.74) | 3.18 (2.88, 3.68) | 0.8*^3^* |
| MESOR | 0.63 (0.61, 0.66) | 0.63 (0.59, 0.67) | 0.60 (0.57, 0.66) | 0.2*^3^* |
| Amplitude | 0.44 (0.40, 0.46) | 0.41 (0.36, 0.45) | 0.43 (0.40, 0.44) | 0.14*^3^* |
| IS | 0.87 (0.83, 0.90) | 0.83 (0.78, 0.89) | 0.88 (0.80, 0.91) | 0.2*^3^* |
| IV | 0.28 (0.23, 0.33) | 0.31 (0.28, 0.37) | 0.30 (0.26, 0.35) | 0.084*^3^* |
| RA | 0.82 (0.76, 0.90) | 0.79 (0.70, 0.87) | 0.84 (0.78, 0.89) | 0.3*^3^* |
| OSAS |  |  |  | **0.015***^5^* |
| *Low* | 2 (8.7%) | 3 (14%) | 24 (39%) |  |
| *Mild* | 14 (61%) | 15 (68%) | 22 (36%) |  |
| *Moderate* | 7 (30%) | 3 (14%) | 12 (20%) |  |
| *High* | 0 (0%) | 1 (4.5%) | 3 (4.9%) |  |
| ^1^BS: Behçet Syndrome; BMI: Bodi Mass Index (kg/m2); PSQI: Pittsburgh Sleep Quality Index; Poor sleeper: PSQI > 5; TST: Total Sleep Time (hours); WASO: Wake After Sleep Onset (minutes); SE: Sleep Efficiency (%); SRI: Sleep Regularity Index (%); rMEQ: reduced Morningness Eveningness Questionnaire; IS: Interdaily Stability; IV: Interaily Variability; RA: relative amplitude; MESOR: Midline-estimating statistic of rhythm  ^2^Median (Q1, Q3); n (%); ^3^ Wilcoxon rank sum test; ^4^ Fisher’s exact test | | | | |

| **SUPPLEMENTARY TABLE S3 Differences in sociodemographic data and sleep and circadian parameters between active BS patients, non-active BS patients and healthy controls (HCs)** | | | | |
| --- | --- | --- | --- | --- |
| **Variable***^1^* | **Active BS patients** N = 12*^2^* | **Non-active BS patients** N = 33*^2^* | **HCs** N = 61*^2^* | **p** |
| Age | 45 (38, 49) | 50 (45, 62) | 49 (36, 58) | 0.12*^3^* |
| BMI | 23.7 (22.3, 26.1) | 24.2 (22.2, 27.8) | 23.8 (22.4, 27.1) | >0.9*^3^* |
| Sex (Female) | 6 (50%) | 24 (73%) | 36 (59%) | 0.3*^4^* |
| Smokers | 1 (8.3%) | 3 (9.1%) | 11 (18%) | 0.6*^4^* |
| Smoking intensity | 0 (0, 0) | 0 (0, 0) | 0 (0, 0) | 0.4*^3^* |
| PSQI | 11.0 (7.5, 13.5) | 8.0 (5.0, 10.0) | 5.0 (3.0, 7.0) | **<0.001***^3^* |
| Poor Sleepers | 10 (83%) | 24 (73%) | 22 (36%) | **<0.001***^4^* |
| TST | 6.43 (6.20, 6.82) | 7.05 (6.30, 7.68) | 7.18 (6.44, 7.70) | 0.2*^3^* |
| SE | 89.8 (82.7, 90.8) | 93.9 (89.3, 95.4) | 94.0 (90.6, 96.1) | **0.004***^3^* |
| WASO | 44 (36, 76) | 29 (22, 43) | 27 (20, 44) | **0.016***^3^* |
| SRI | 74 (64, 79) | 78 (73, 83) | 81 (70, 84) | 0.2*^3^* |
| rMEQ | 14 (12, 18) | 17 (15, 18) | 16 (15, 19) | 0.2*^3^* |
| Acrophase | 15.22 (14.73, 15.61) | 15.70 (14.92, 16.15) | 15.53 (14.90, 16.17) | 0.3*^3^* |
| Mid-sleep point | 3.07 (2.56, 3.47) | 3.27 (2.77, 3.81) | 3.18 (2.88, 3.68) | 0.6*^3^* |
| MESOR | 0.66 (0.62, 0.68) | 0.63 (0.61, 0.66) | 0.60 (0.57, 0.66) | 0.15*^3^* |
| Amplitude | 0.42 (0.36, 0.46) | 0.42 (0.40, 0.46) | 0.43 (0.40, 0.44) | >0.9*^3^* |
| IS | 0.88 (0.85, 0.91) | 0.84 (0.80, 0.89) | 0.88 (0.80, 0.91) | 0.4*^3^* |
| IV | 0.30 (0.25, 0.34) | 0.30 (0.26, 0.35) | 0.30 (0.26, 0.35) | 0.8*^3^* |
| RA | 0.78 (0.67, 0.83) | 0.84 (0.75, 0.89) | 0.84 (0.78, 0.89) | 0.15*^3^* |
| OSAS |  |  |  | **<0.001***^4^* |
| *Low* | 3 (25%) | 2 (6.1%) | 24 (39%) |  |
| *Mild* | 4 (33%) | 25 (76%) | 22 (36%) |  |
| *Moderate* | 4 (33%) | 6 (18%) | 12 (20%) |  |
| *High* | 1 (8.3%) | 0 (0%) | 3 (4.9%) |  |
| ^1^BS: Behçet Syndrome; BMI: Bodi Mass Index (kg/m2); PSQI: Pittsburgh Sleep Quality Index; Poor sleeper: PSQI > 5; TST: Total Sleep Time (hours); WASO: Wake After Sleep Onset (minutes); SE: Sleep Efficiency (%); SRI: Sleep Regularity Index (%); rMEQ: reduced Morningness Eveningness Questionnaire; IS: Interdaily Stability; IV: Interaily Variability; RA: relative amplitude; MESOR: Midline-estimating statistic of rhythm. ^2^Median (Q1, Q3); n (%); ^3^ Wilcoxon rank sum test; ^4^ Fisher’s exact test | | | | |

| **SUPPLEMENTARY TABLE S4 Linear Regression Models Investigating Possible Associations between Active disease, Fibromyalgia, and Sleep and Circadian Parameters** | | | | | | | | | |
| --- | --- | --- | --- | --- | --- | --- | --- | --- | --- |
| **Independent Variables** | **Active Disease** | **Fibromyalgia** | **Age** | **BMI** | **Sex** | **Smoker** | **Mood Disorders/**  **Insomnia Treatment** | **R²** | **Adj-R²** |
| PSQI | 3.2 (**<0.001*****) | 4.1 (**<0.001*****) | 0.03 (0.10) | 0.11 (0.11) | -1.0 (0.075) | 0.38 (0.7) | 0.50 (0.7) | 0.455 | 0.416 |
| TST | -0.24 (0.5) | -0.72 (**0.043***) | -0.01 (0.2) | 0.00 (0.9) | -0.21 (0.4) | -0.35 (0.3) | 0.78 (0.080) | 0.08 | 0.015 |
| SE | -4.4 (**0.008****) | -3.9 (**0.015***) | -0.03 (0.5) | -0.16 (0.2) | 0.20 (0.8) | 2.1 (0.2) | 1.4 (0.5) | 0.207 | 0.15 |
| WASO | 19 (**0.017***) | 13 (0.081) | 0.07 (0.7) | 0.52 (0.4) | -2.2 (0.7) | -10 (0.2) | 2.5 (0.8) | 0.173 | 0.114 |
| SRI | -3.6 (0.3) | 0.11 (>0.9) | 0.21 (**0.014***) | -0.45 (0.10) | -1.1 (0.6) | -1.4 (0.7) | 1.1 (0.8) | 0.125 | 0.062 |
| rMEQ | -1.6 (0.10) | 0.08 (>0.9) | 0.06 (**0.014***) | 0.00 (>0.9) | -0.81 (0.2) | -2.3 (**0.013***) | -2.0 (0.10) | 0.229 | 0.174 |
| Acrophase | -0.51 (0.094) | 0.05 (0.9) | -0.01 (**0.043***) | -0.01 (0.7) | -0.19 (0.3) | 0.12 (0.7) | 0.41 (0.3) | 0.102 | 0.037 |
| Mid-sleep point | -0.30 (0.3) | -0.09 (0.7) | -0.01 (0.2) | 0.01 (0.7) | -0.25 (0.14) | 0.34 (0.2) | 0.29 (0.4) | 0.093 | 0.028 |
| Amplitude | -0.01 (0.7) | 0.01 (0.5) | 0.00 (>0.9) | 0.00 (>0.9) | 0.00 (0.7) | 0.02 (0.2) | 0.02 (0.3) | 0.062 | -0.005 |
| MESOR | 0.02 (0.2) | 0.02 (0.3) | 0.00 (0.4) | 0.00 (0.2) | 0.00 (>0.9) | 0.02 (0.2) | -0.02 (0.3) | 0.066 | -0.001 |
| IS | 0.02 (0.5) | 0.04 (0.13) | 0.00 (**0.028***) | 0.00 (0.8) | 0.01 (0.4) | -0.01 (0.6) | -0.02 (0.5) | 0.098 | 0.034 |
| IV | -0.01 (0.6) | -0.02 (0.4) | 0.00 (0.8) | 0.00 (0.3) | 0.01 (0.7) | -0.03 (0.12) | -0.02 (0.4) | 0.092 | 0.028 |
| RA | -0.06 (0.077) | -0.02 (0.6) | 0.00 (0.8) | 0.00 (0.14) | 0.01 (0.7) | 0.03 (0.3) | 0.05 (0.2) | 0.08 | 0.015 |
| ^1^BS: Behçet Syndrome; BMI: Bodi Mass Index (kg/m2); Smoker (yes/no); PSQI: Pittsburgh Sleep Quality Index; TST: Total Sleep Time (hours); WASO: Wake After Sleep Onset (minutes); SE: Sleep Efficiency (%); SRI: Sleep Regularity Index (%); IS: Interdaily Stability; IV: Interaily Variability; RA: relative amplitude; MESOR: Midline-estimating statistic of rhythm; rMEQ: reduced Morningness Eveningness Questionnaire. | | | | | | | | | |

| **SUPPLEMENTARY TABLE S5 Differences in glucocorticoids (GC) exposure variables and comorbid fibromyalgia prevalence across different OSAS risk categories in the whole sample (N=106)** | | | | | | | | |
| --- | --- | --- | --- | --- | --- | --- | --- | --- |
| **Variable***^1^* | **Low^A^** N = 29*^2^* | **Mild^B^** N = 51*^2^* | **Moderate-to-high^C^** N = 26*^2^* | **p***^3^* | **p^A,C^** | **p^B,C^** | **p^A,B^** |  |
| GC | 1 (3.4%) | 11 (22%) | 6 (23%) | 0.053 | **0.044*** | 0.9 | **0.047*** |  |
| dGC | 0.0 (0.0, 0.0) | 0.0 (0.0, 5.0) | 2.5 (0.0, 5.0) | 0.5 | 0.3 | 0.5 | 0.5 |  |
| One-year cumulative GC dose | 0 (0, 0) | 0 (0, 168) | 0 (0, 360) | **0.016*** | **0.004**** | 0.6 | **0.010*** |  |
| Fibromyalgia | 2 (6.9%) | 14 (27%) | 7 (27%) | 0.076 | 0.069 | >0.9 | **0.027*** |  |
| Possible differences between couples of OSAS risk categories are specified by footnotes A (low), B (mild), and C (moderate-to-high). | | | | | | | | |
| *^1^*GC: glucocorticoids (GC) use at the time of recruitment, dGC: daily GC dose (mg, prednisone equivalents).  *^2^* n (%); Median (Q1, Q3)  *^3^* Kruskal-Wallis rank sum test; Fisher’s exact test; Wilcoxon rank-sum test | | | | | | | | |

## Supplementary Figure S1


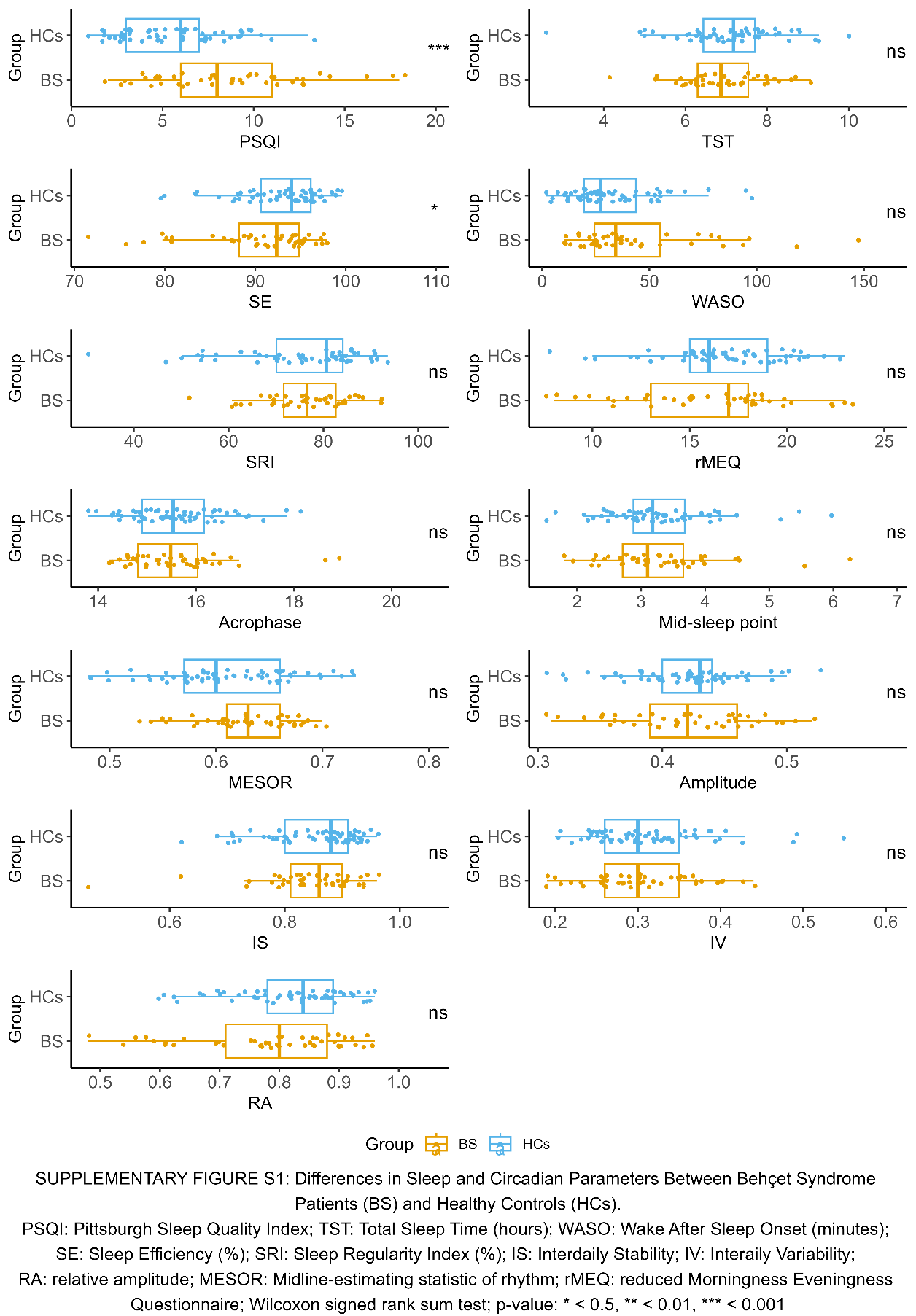


## Supplementary Figure S2


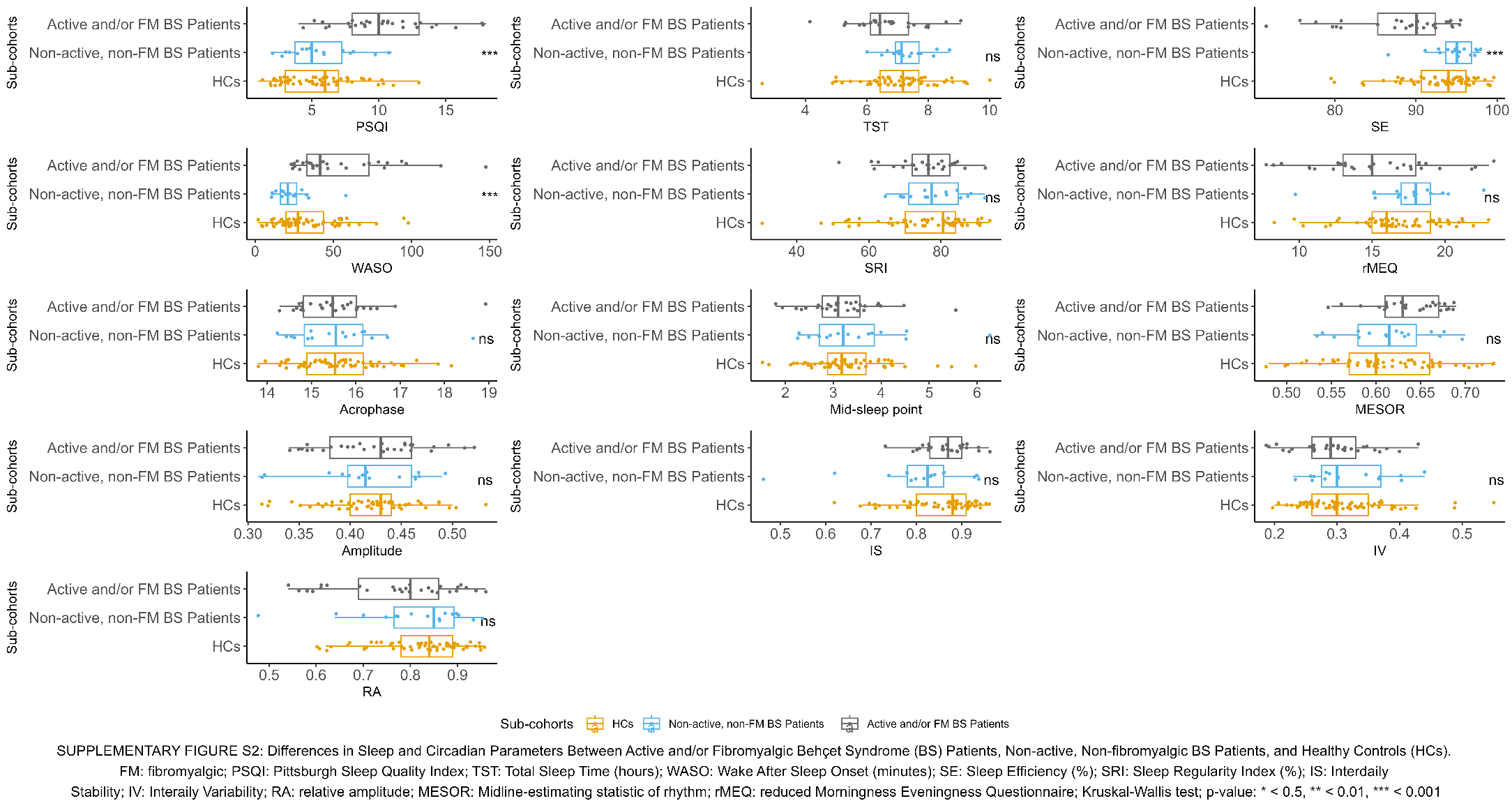

Supplement: keaf326_Supplementary_Data [file keaf326_supplementary_data.zip › keaf326_Supplementary_Data/rhe-25-0841-File003.docx]
